# Tunning CO_2_ Separation Performance of Ionic Liquids through Asymmetric Anions

**DOI:** 10.3390/molecules27020413

**Published:** 2022-01-09

**Authors:** Bruna F. Soares, Daniil R. Nosov, José M. Pires, Andrey A. Tyutyunov, Elena I. Lozinskaya, Dmitrii Y. Antonov, Alexander S. Shaplov, Isabel M. Marrucho

**Affiliations:** 1 Centro de Química Estrutural, Departamento de Engenharia Química, Institute of Molecular Sciences, Instituto Superior Técnico de Lisboa, Universidade de Lisboa, Av. Rovisco Pais 1, 1049-001 Lisboa, Portugal; bruna.soares@tecnico.ulisboa.pt (B.F.S.); jose.m.pires@tecnico.ulisboa.pt (J.M.P.); 2 Luxembourg Institute of Science and Technology (LIST), 5 Avenue des Hauts-Fourneaux, L-4362 Esch-sur-Alzette, Luxembourg; daniil.nosov@list.lu (D.R.N.); alexander.shaplov@list.lu (A.S.S.); 3 Department of Physics and Materials Science, University of Luxembourg, 2 Avenue de l’Université, L-4365 Esch-sur-Alzette, Luxembourg; 4 A.N. Nesmeyanov Institute of Organoelement Compounds Russian Academy of Sciences (INEOS RAS), Vavilov Str. 28, GSP-1, 119991 Moscow, Russia; tuytuynov@rambler.ru (A.A.T.); helloz@ineos.ac.ru (E.I.L.); dyuant@ineos.ac.ru (D.Y.A.)

**Keywords:** gas separation, ionic liquids, carbon dioxide, supported liquid membranes, asymmetric anions

## Abstract

This work aims to explore the gas permeation performance of two newly-designed ionic liquids, [C_2_mim][CF_3_BF_3_] and [C_2_mim][CF_3_SO_2_C(CN)_2_], in supported ionic liquid membranes (SILM) configuration, as another effort to provide an overall insight on the gas permeation performance of functionalized-ionic liquids with the [C_2_mim]^+^ cation. [C_2_mim][CF_3_BF_3_] and [C_2_mim][CF_3_SO_2_C(CN)_2_] single gas separation performance towards CO_2_, N_2_, and CH_4_ at T = 293 K and T = 308 K were measured using the time-lag method. Assessing the CO_2_ permeation results, [C_2_mim][CF_3_BF_3_] showed an undermined value of 710 Barrer at 293.15 K and 1 bar of feed pressure when compared to [C_2_mim][BF_4_], whereas for the [C_2_mim][CF_3_SO_2_C(CN)_2_] IL an unexpected CO_2_ permeability of 1095 Barrer was attained at the same experimental conditions, overcoming the results for the remaining ILs used for comparison. The prepared membranes exhibited diverse permselectivities, varying from 16.9 to 22.2 for CO_2_/CH_4_ and 37.0 to 44.4 for CO_2_/N_2_ gas pairs. The thermophysical properties of the [C_2_mim][CF_3_BF_3_] and [C_2_mim][CF_3_SO_2_C(CN)_2_] ILs were also determined in the range of T = 293.15 K up to T = 353.15 K at atmospheric pressure and compared with those for other ILs with the same cation and anion’s with similar chemical moieties.

## 1. Introduction

Although Ionic Liquids (ILs) were first introduced as a green alternative to conventional organic solvents, mainly due to their vanishing vapor pressure [1], it was their unusual set of properties, such as low volatility, high thermal stability, low flammability [2,3] together with the possibility to tune the ILs properties through the combination of endless number of cations and anions [4], that afforded their popularity. Today, it is well known that ILs are not intrinsically green, not only due to their fossil fuel source, but also their complex, expensive, and non-sustainable synthesis, purification processes, and their recalcitrant nature. Nevertheless, ILs can provide the implementation of more sustainable processes, being at the center of clever solutions to common problems.

Ionic liquid-based membranes have won the interest of the scientific community since they gather the benefits of membrane separation, together with the unique characteristics of ILs. Several studies show the feasibility of using these membranes at an industrial scale to capture CO_2_ from flue gases [5] to purify natural gas [6], in the recovery and enrichment of biohydrogen [7], to separate hydrogen from ammonia purge gas [8], to obtain oxygen or inert gases, and to separate volatile organic compounds (VOCs) from gas streams [9]. Among the different membrane configurations, Supported Ionic Liquid Membranes (SILMs) are a very attractive technology due to their high gas permeabilities, the high gas solubility characteristic of a liquid phase, the small amount of IL required, and the easiness of fabrication. On top of that, the negligible vapor pressure of ILs is a real advantage since it eliminates the risk of losing the sorbent fluid through evaporation. The only requirement is chemical compatibility of the external porous support and the IL, for which properties such as viscosity, surface tension, and surface free energy play a relevant role. However, SILMs industrial relevance is restricted to low operation pressures and temperatures, since otherwise the IL is pushed out of the pores, leading to membrane instability [10]. However, recent pilot-scale trials at power plants in Germany showed that optimized SILMs could maintain their structural and process stability for 335 h [11]. The success of SILMs is well highlighted in recently published reviews [4,12,13,14,15,16,17].

SILMs are also an excellent tool for more fundamental research studies of the relevance of IL chemical structures in specific gas separation applications. The great versatility of ILs chemical structures, through the combination of different cations and anions, has been thoroughly researched in SILMs. It has been shown that the anion plays a more relevant role in gas separation than the cation [18,19,20]. Consequently, most of the studies relate to 1-ethyl-3-methyl, [C_2_mim]^+^, or 1-butyl-3-methyl imidazolium, [C_4_mim]^+^, cations, although some reports addressed the effect of imidazolium cation modifications, such as the introduction of long alkyl side chains or functionalized alkyl side chains. However, only a limited number of publications are devoted to the investigation of ILs having other cations, namely ammonium, in particular cholinium, and phosphonium [21,22,23,24,25,26,27]. 

In another vein, IL-IL mixtures have also shown to provide a further increase in flexibility and the fine-tune capacity of the physical and chemical properties of these compounds, providing an extra degree of freedom in the design of new fillers for SILMs. Several studies regarding the effect of the IL-IL mixture have been recently reported [28,29,30]. Since most IL-IL mixtures exhibit an almost ideal behavior, the precise tunning of physical chemical properties can be generally done, such as the case of the mixtures of [C_2_mim][Ac], [C_2_mim][Lac], [C_2_mim][C(CN)_2_], [C_2_mim][SCN] with [C_2_mim][NTf_2_], where no synergist effect between the ILs was observed. However, mixing ILs with the common [C_2_mim]^+^ cation and different anions containing cyano groups, namely thiocyanate [SCN]^−^, dicyanamide [N(CN)_2_]^−^, tricyanomethane [C(CN)_3_]^−^, and tetracyanoborate [B(CN)_4_]^−^ anions, a non-linear behavior was found in CO_2_, CH_4_, and N_2_ permeation properties, affording an increase of more than 200% in CO_2_ permeability for some of these mixtures [29]. Nevertheless, (CO_2_/N_2_) and (CO_2_/CH_4_) selectivity values were in between those of the neat ILs. 

The comparison of SILMs filled with 1-ethyl-3-methyl imidazolium ILs with different anions shows that the highest CO_2_ permeability and diffusivity were achieved for ILs with the lowest viscosity [31,32]. Thus, the improvement of SILMs characteristics can be further achieved via the development of novel anions that will afford ILs with low viscosity. One possibility to reduce the viscosity of ILs is the introduction of asymmetry in the anion’s structure, a strategy that was firstly employed by Matsumoto et al. [33,34] when the 2,2,2-trifluoro-N-(trifluoromethylsulfonyl)acetimide, [TSAC]^−^ anion was introduced. [TSAC]^−^ anion demonstrated an excellent ability to decrease both the melting points and the viscosities of its salts containing even small aliphatic ammonium cations such as N,N-dimethylpyrrolidinium, which could not form a room temperature ILs with other known symmetrical anions like [BF_4_]^−^ or [NTf_2_]^−^. This principle was further applied in the design of new asymmetric anions, namely CF_3_SO_2_-N-CN [TFSAM] [35], CF_3_SO_2_-N-S(CF_3_)=O [36], CF_3_-SO(NSO_2_CF_3_)-N-SO_2_CF_3_ [37], CF_3_SO_2_-N-SO_2_F [FTFSI] or [FTA] [38], C_2_F_5_SO_2_-N-SO_2_F [FPFSI] [39], CF_3_SO_2_-N-SO(CF_3_)=N-SO_2_CF_3_ [40], R-BF_3_ (R = CnH_2_n+1, n = 1–5) [41], Rf-BF_3_ (Rf = CnF_2_n+1, n = 1–4) [42], and the synthesis of ILs on their basis. Taking this into account, in a recent work [43,44], we compared the gas permeation performance of SILMs filled with low viscous [C_2_mim][TFSAM] with those saturated with an equimolar mixture containing [C_2_mim][NTf_2_] and [C_2_mim][C(CN)_2_]. The results demonstrated that SILM with neat [C_2_mim][TFSAM] displays higher CO_2_ permeability, diffusivity, and solubility than SILMs with the selected ILs mixtures. Although, it should be mentioned that the CO_2_/N_2_ selectivity of both SILMs was similar and can be positioned on top of or slightly above the Robeson plot [45]. 

**Scheme 1:**
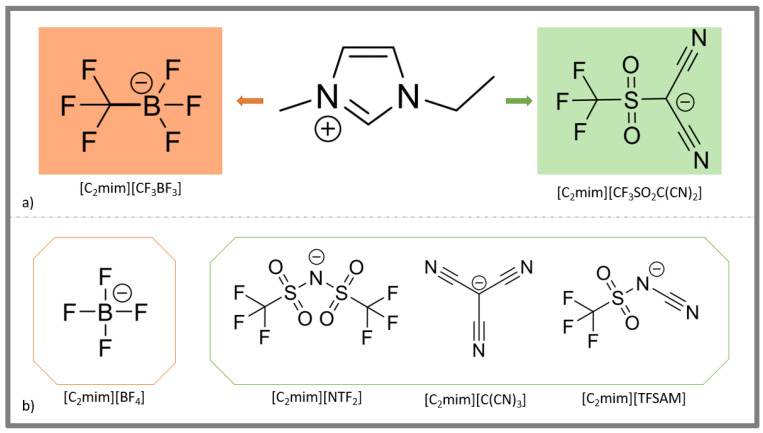
Chemical structures of ionic liquids (ILs) studied in this work [C_2_mim][CF_3_BF_3_] and [C_2_mim][CF_3_SO_2_C(CN)_2_] (**a**); and of other ILs used for comparison (**b**) namely [C_2_mim][BF_4_], [C_2_mim][NTf_2_], [C_2_mim][C(CN)_3_], and [C_2_mim][TFSAM].

**Scheme 2:**
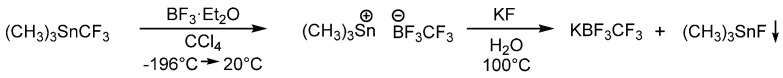
Synthetic pathway for the preparation of K[CF_3_BF_3_] according to Chambers, Clark, and Willis [48].

In this work, we step forward in the design of new asymmetrical anions (Scheme 1). Two anions, namely trifluoro(trifluoromethyl)borate [CF_3_BF_3_]^−^ and dicyano((trifluoromethyl)sulfonyl)methanide [DCATfM]^−^ or [CF_3_SO_2_C(CN)_2_]^−^, were prepared in the form of their alkali salts and used for the synthesis of [C_2_mim]^+^ containing Ils. While [DCATfM]- anion was synthesized for the first time, for the preparation of K[BF_3_CF_3_] salt, we propose an improved approach in comparison with literature procedure [46], which avoids the use of corrosive hydrogen fluoride and toxic hexamethyltin (Scheme 2). Both [C_2_mim][CF_3_BF_3_] and [C_2_mim][CF_3_SO_2_C(CN)_2_] ILs show low viscosity values equal to 25.59 and 25.00 mPa·s at 298.15 K, respectively. The obtained viscosity values seem to be relatively low, since typically imidazolium-based ILs show dynamic viscosities in the range between 0.841 and 2.57 × 10^5^ mPa·s, as shown in recent works on the predictive estimation of pure ILs viscosities [47]. They were further used in the preparation of new SILMs and study of their gas separation properties. 

## 2. Results and Discussion

### 2.1. Synthesis of New ILs

Potassium (trifluoromethyl)trifluoroborate K[CF_3_BF_3_] is an air- and water-stable solid that was first reported in 1960 by Chambers, Clark, and Willis [48]. The synthesis was carried out in two steps (Scheme 2). On the first step, the trimethyltrifluoromethyltin reacted with boron trifluoride with the formation of a 1:1 adduct, that precipitated immediately from the tetrachloride solution. The second step consisted of the ion exchange with potassium fluoride in an aqueous medium, where the insoluble trimethyltin fluoride was precipitated and filtered off, while the evaporation of the resulting solution provided potassium trifluoromethylfluoroborate. Despite the good purity of obtained K[CF_3_BF_3_], the use of this method is limited by the high toxicity of the tin compounds. 

**Scheme 3:**
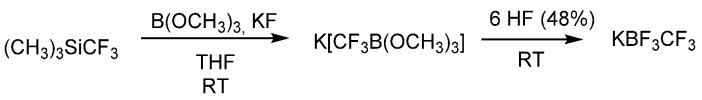
Synthetic pathway for the preparation of K[CF_3_BF_3_] following Molander [46].

Later on, in 2003, Molander [46] suggested an improved method for the preparation of K[CF_3_BF_3_]. Instead of toxic trimethyltrifluoromethyltin, it was suggested to start the reaction with Ruppert’s reagent, (trifluoromethyl)trimethylsilane, which was treated with trimethoxyborane in the presence of potassium fluoride (Scheme 3). Lately, the aqueous hydrogen fluoride was added to the resulting intermediate, and the title compound was isolated in 85% overall yield. Although, both the yield and purity of K[CF_3_BF_3_] were sufficiently high, this method suffered from the usage of corrosive hydrogen fluoride and thus, from the impossibility to carry out the reaction in glass reactors and the necessity for special vessels made of copper or Teflon. 

**Scheme 4:**
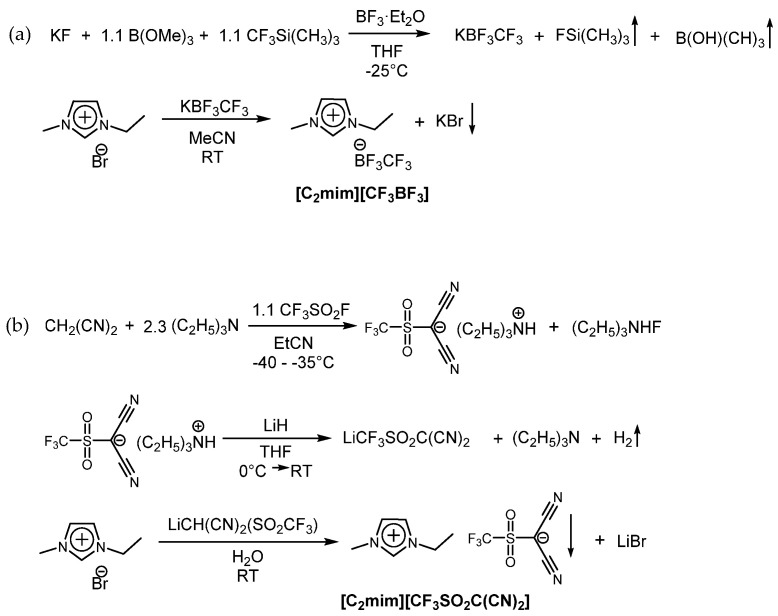
Reaction pathway for the synthesis of [C_2_mim][CF_3_BF_3_] (**a**) and [C_2_mim][CF_3_SO_2_C(CN)_2_] (**b**) used in this study.

In this work, we introduced a new method for the synthesis of K[CF_3_BF_3_] (Scheme 4). As in the Molander approach [46], the first step consisted in the formation of so-called “ate” complex, although the subsequent fluorination with hydrogen fluoride was replaced with a substitution with BF_3_·Et_2_O, followed by the removal of B(OCH_3_)_3_ by distillation. Thus, the suggested approach differs from the previous ones [46,48] by the absence of toxic tin compounds as well as the substitution of corrosive HF with BF_3_·Et_2_O. The prepared K[CF_3_BF_3_] salt was used for the synthesis of [C_2_mim][CF_3_BF_3_] via metathesis reaction (Scheme 4). As [C_2_mim][CF_3_BF_3_] was found to be soluble in water at average concentrations, an ion exchange reaction of K[CF_3_BF_3_] with [C_2_mim][Br] was performed in anhydrous acetonitrile. After the filtration of precipitated KBr and evaporation of solvent, the [C_2_mim][CF_3_BF_3_] was obtained as a colorless oil. 

The Li[CF_3_SO_2_C(CN)_2_] was synthesized in two steps (Scheme 4). On the first step, malonitrile was deprotonated with triethylamine and further reacted with trifluoromethanesulphonyl fluoride at −40 °C. The isolated triethylammonium dicyano((trifluoromethyl)sulfonyl)methanide further reacted with lithium hydride in polar THF to form Li[CF_3_SO_2_C(CN)_2_]. The ion exchange reaction with [C_2_mim][Br] was carried out in water, with precipitation of the desired [C_2_mim][CF_3_SO_2_C(CN)_2,_] with 92% yield.

The structure and purity of [C_2_mim][CF_3_BF_3_] and [C_2_mim][CF_3_SO_2_C(CN)_2_] ILs were proved by ^1^H, ^13^C, ^11^B, ^19^F NMR, and IR spectroscopy as well as by elemental analysis. For [C_2_mim][CF_3_BF_3_], the ^19^F NMR displayed two multiplets at −153.7 and −73.8 ppm that were attributed to BF_3_ and CF_3_, respectively. ^11^B NMR showed quadruplet of quadruplets at −1.44 ppm. FTIR spectra of [C_2_mim][CF_3_BF_3_] demonstrated the characteristic absorption bands at 3171, 3128 and 2990 (CH stretching), 1057 and 634 (B–F vibrations), and at 952 cm^−1^ (CF vibrations). The ^19^F NMR of [C_2_mim][CF_3_SO_2_C(CN)_2_] showed a singlet at −81.5 ppm assigned to CF_3_ group. Both CF_3_ and CN groups were found in ^13^C NMR as a quadruplet at 125.6–115.9 and as singlet at 115.5 ppm, respectively. FTIR spectra of [C_2_mim][CF_3_SO_2_C(CN)_2_] contained the following characteristic absorption bands: 3158, 3119 and 2990 cm^−1^ attributed to CH stretching; 1350 and 1181 cm^−1^ assigned to asymmetric and symmetric vibrations of S=O bond; and finally 1209 and 1071 cm^−1^ bands that were designated to the CF vibrations. 

### 2.2. Thermal Properties of ILs

Thermal properties of [C_2_mim][CF_3_BF_3_] and [C_2_mim][CF_3_SO_2_C(CN)_2_] ILs were studied by DSC and TGA. The DSC of [C_2_mim][CF_3_BF_3_] revealed two transitions, namely the melting point and crystallization at 255.1 K (*T*_m_) and 201.1 K (*T*_cr_), respectively. These values are in good agreement with those published by Zhou and Matsumoto, 253 K and 193 K [42], respectively. The small difference is probably caused by the difference in scanning rates (2 and 10 K min^−1^ in this study and in [42], correspondingly). Upon the comparison of melting points for [C_2_mim][CF_3_BF_3_] and its structural analogue [C_2_mim][BF_4_] (*T*_m_ = 286–288 K [49]) it becomes obvious that the asymmetry of [CF_3_BF_3_] anion allows to decrease the *T*_m_ of IL by nearly 303 K.

The DSC traces of [C_2_mim][CF_3_SO_2_C(CN)_2_] performed even at a slow rate of 2 Kmin^−1^, recommended for accurate measurements of thermal properties for ILs [50,51], confirmed the absence of any crystallization or melting processes. It was revealed, that in contrast to [C_2_mim][C(CN)_3_] IL with symmetrical tricyanomethanide anion, which possesses both the *T*_m_ = 262 K and *T*_cr_ = 213 K [52], the novel [C_2_mim][CF_3_SO_2_C(CN)_2_] shows only the glass transition temperature *T*_g_ at 188.9 K. 

The thermal degradation behavior of ILs was assessed by TGA in air. The weight loss profiles of both [C_2_mim][CF_3_BF_3_] and [C_2_mim][CF_3_SO_2_C(CN)_2_] compounds revealed a one-step degradation mechanism. The determined onset weight loss temperatures were found to be 488 K and 573 K for [C_2_mim][CF_3_BF_3_] and [C_2_mim][CF_3_SO_2_C(CN)_2_], respectively. Comparing these temperatures with those of other ILs with similar anions such as [C_2_mim][BF_4_] and [C_2_mim][NTf_2_], the present thermal decomposition temperatures of 713.15 K [53] and 668.15 K [54], it can be concluded that these new ILs show a loss thermal stability.

### 2.3. Termophysical Properties of ILs: Density, Molar Volume, and Viscosity

**Figure 1:**
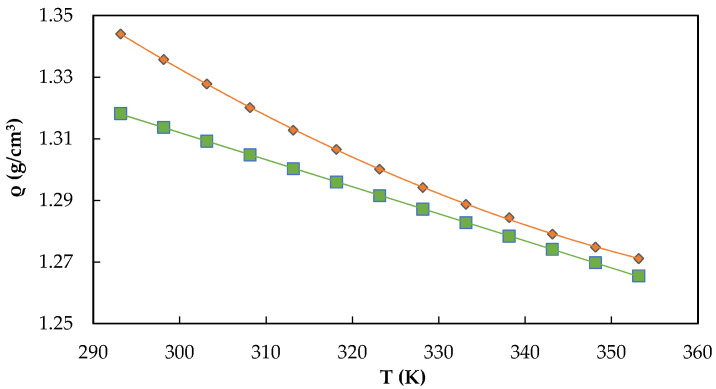
Temperature dependence of density (ρ, g·cm^−3^) for [C_2_mim][CF_3_BF_3_] (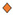) and [C_2_mim][CF_3_SO_2_C(CN)_2_] (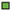). The errors bars are smaller than the symbols used to represent the experimental data.

Density and viscosity are very important properties of ILs used in gas separation since they have a direct influence on gas permeation properties. Both density and viscosity for [C_2_mim][CF_3_BF_3_] and [C_2_mim][CF_3_SO_2_C(CN)_2_] were measured within the range of 293.15 K up to 353.15 K at ambient pressure and their behaviour with temperature is presented in Figure 1. The values are listed in Table S1 in Supporting Information.

From Figure 1 it is possible to see that the temperature dependence of density for [C_2_mim][CF_3_BF_3_] is significantly different from that of [C_2_mim][CF_3_SO_2_C(CN)_2_]. While [C_2_mim][CF_3_BF_3_] shows a quadratic dependence, [C_2_mim][CF_3_SO_2_C(CN)_2_] demonstrates a nearly linear dependence. In the studied temperature range, the [C_2_mim][CF_3_BF_3_] possessed higher densities when compared to those of [C_2_mim][CF_3_SO_2_C(CN)_2_]. However, as the temperature increases, this difference becomes less pronounced and was reaching very close values at 353.15 K, and 1.271 and 1.266 g cm^−3^ for [C_2_mim][CF_3_BF_3_] and [C_2_mim][CF_3_SO_2_C(CN)_2_], respectively.

*Ρ* = *a* − *bT* + *cT*^2^ (1)

where *T* is temperature in K and *a*, *b*, and *c* are adjustable parameters, which are listed in Table 1. For [C_2_mim][CF_3_BF_3_], a polynomial second-order fitting [55,56] was used, while for [C_2_mim][CF_3_SO_2_C(CN)_2_] a linear equation was well describing the experimental data (*c* = 0), as it can be observed from the correlation coefficient R^2^ listed in Table 1.

The isobaric thermal expansion coefficients (α_P_) were calculated using density experimental data through Equation (2).

(2) $$\alpha_{P}=-\frac{1}{\rho }{\left(\frac{\partial \rho }{\partial T}\right)}_{P}$$

where *ρ* is the density in g·cm^−3^ and *P* is the pressure. The thermal expansion coefficient values of [C_2_mim][CF_3_BF_3_] and [C_2_mim][CF_3_SO_2_C(CN)_2_] are provided in Table S3. For [C_2_mim][CF_3_BF_3_] they varied from 9.06 × 10^−4^ to 9.58 × 10^−4^ K^−1^ in the 293.15–353.15 K temperature range, while for [C_2_mim][CF_3_SO_2_C(CN)_2_] much lower values were obtained, ranging from 6.67 × 10^−4^ to 6.94 × 10^−4^ K^−1^. When comparing these results with those obtain for other ILs, it can be concluded that the thermal expansion coefficients for [C_2_mim][CF_3_SO_2_C(CN)_2_] are similar to those of other ILs, between 5.86 × 10^−4^ K^−1^ for [C_2_mim][BF_4_] and 6.42 × 10^−4^ K^−1^ for [C_2_mim][NTf_2_]. However, for the [C_2_mim][CF_3_BF_3_] IL, much higher (50% higher) thermal expansion coefficients were measured, indicating a larger change in volume with temperature compared to other ILs.

The molar volumes *V*_M_ (cm^3^·mol^−1^) were also calculated for the two studied Ils, in the same range of temperatures using Equation (3).

(3) $$V_{M}=\frac{M}{\rho }$$

where *ρ* corresponds to the density (g·cm^−3^) and *M* is the molar mass (g·mol^−1^). The calculated molar volumes are listed in Table S2 and represented in Figure S1. As expected, *V*_M_ values for [C_2_mim][CF_3_BF_3_] were higher than those of [C_2_mim][CF_3_SO_2_C(CN)_2_] in the whole temperature range.

**Figure 2:**
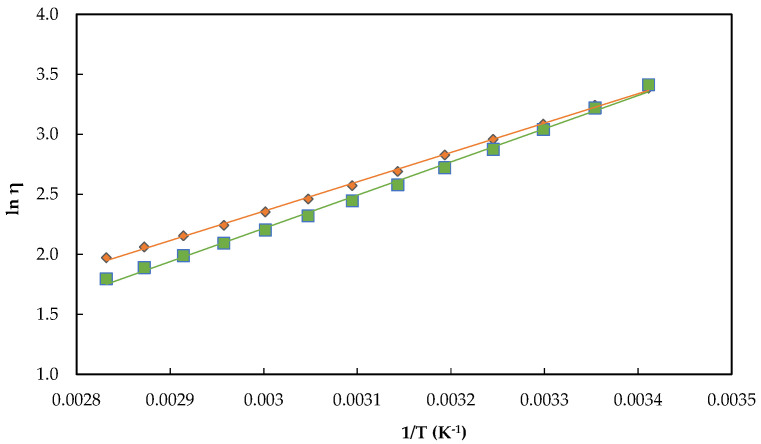
Temperature dependence of the logarithmic experimental viscosities (η) for [C_2_mim][CF_3_BF_3_] (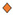) and [C_2_mim][CF_3_SO_2_C(CN)_2_] (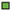). The errors bars marked are smaller than the symbols used to represent the experimental data.

The viscosity measurements of [C_2_mim][CF_3_BF_3_] and [C_2_mim][CF_3_SO_2_C(CN)_2_] in the temperature range between 293.15 K and 353.15 K and at atmospheric pressure are listed in Table S4 and depicted in Figure 2.

Both [C_2_mim][CF_3_BF_3_] and [C_2_mim][CF_3_SO_2_C(CN)_2_] Ils present very similar viscosity values in the low temperature region. For example, at 293.15 K, a viscosity of 29.47 mPa·s was obtained for [C_2_mim][CF_3_BF_3_], while for [C_2_mim][CF_3_SO_2_C(CN)_2_ a value of 30.35 mPa·s was measured. Moreover, as the temperature increases, both values tend to slowly depart. Equation (4) was used to describe the temperature behavior of viscosity.

(4) $$\ln\eta =\ln\eta_{\infty }-\frac{E_{a}}{RT}\;$$

where *η* corresponds to the viscosity in mPa·s, *E_a_* is the activation energy, *R* is the ideal gas constant, and *T* is the temperature in K. The values *η_∞_* and *E_a_* for the two ILs under study are listed in Table 2.

The activation energy can be interpreted as the energy barrier of a fluid to shear stress. The higher the *E_a_* value, the more difficult it is for the molecules/aggregates/ion pairs to move past each other. This can be a direct consequence of the size or entanglement of the molecules/aggregates/ion pairs and/or the presence of stronger interactions within the IL. It can be observed that [C_2_mim][CF_3_BF_3_] presents a lower *E_a_* value than the [C_2_mim][CF_3_SO_2_C(CN)_2_], although they both possess similar cation. In its turn, this means that [C_2_mim][CF_3_SO_2_C(CN)_2_] is more sensitive to changes in viscosity when there is an increase of temperature.

As mentioned in the introduction, it is interesting to compare the density and viscosity values obtained for the two ILs under study, [C_2_mim][CF_3_BF_3_] and [C_2_mim][CF_3_SO_2_C(CN)_2_], with those of other ILs with the same cation and similar chemical moieties in the anion. In particular, the properties of [C_2_mim][CF_3_SO_2_C(CN)_2_] can be compared with those of [C_2_mim][TFSAM], with a smaller asymmetrical anion, and with [C_2_mim][NTf_2_] and [C_2_mim][C(CN)_3_], having symmetrical anions. On the other hand, the properties of [C_2_mim][CF_3_BF_3_] can be directly compared with those of [C_2_mim][BF_4_]. Table 3 compares density, viscosity, and molar volumes of several ILs relevant to this work at 298.15 K.

The comparison of [C_2_mim][CF_3_BF_3_] density with that of [C_2_mim][BF_4_] allows to conclude that the introduction of the CF_3_ group in the [BF_4_]^−^ anion leads to a significant increase in density, and consequently in the molar volume (Table 3). This is in agreement with the increase in density of ILs from [C_2_mim][NTf_2_] to [C_2_mim][N(SO_2_C_2_F_5_)] or, in other words, upon the increase in the number of CF_3_ groups in the anion. It can be observed that the density of [C_2_mim][NTf_2_] is significantly higher than that of [C_2_mim][N(CN)_2_] leading to the conclusion that the insertion of the CN group into the imide anion leads to the reduction of the overall density of ILs. Indeed, the density value for [C_2_mim][TFSAM], with the asymmetrical anion containing one CF_3_SO_2_ and one CN group, can be placed exactly in between the densities of the “parent” [C_2_mim][NTf_2_] and [C_2_mim][N(CN)_2_] ILs (Table 3). Similarly, the substitution of one CN group by the CF_3_SO_2_ group in the [C(CN)_3_]^-^ anion and the formation of the [CF_3_SO_2_C(CN)_2_]^-^ anion, resulted in the increase of [C_2_mim] [CF_3_SO_2_C(CN)_2_] density in comparison with [C_2_mim][C(CN)_3_].

Similar trends were found to be correct for the viscosity dependence (Table 3) as the introduction of CF_3_SO_2_ and CF_3_ groups in the anions structure leads to an increase in the viscosity, while the insertion of CN groups has the opposite behavior. Thus, the viscosity of [C_2_mim][CF_3_BF_3_] was found to be higher than that of [C_2_mim][BF_4_], though the viscosity of [C_2_mim][CF_3_SO_2_C(CN)_2_] is in between those of [C_2_mim][NTf_2_] and [C_2_mim][C(CN)_3_]. 

### 2.4. Gas Permeability, Diffusivity, and Solubility of CO_2_, CH_4_, and N_2_ in SILMs

**Figure 3:**
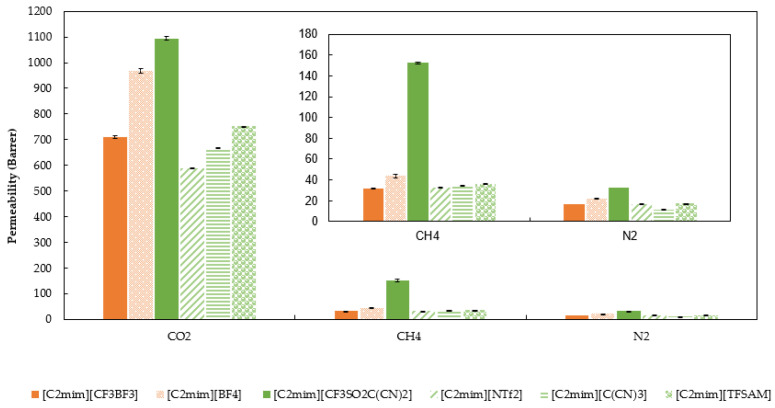
CO_2_, N_2_, and CH_4_ gas permeabilities (P) through [C_2_mim][CF_3_BF_3_] and [C_2_mim][CF_3_SO_2_C(CN)_2_] SILMs at T = 20 °C. Error bars represent standard deviations based on three experimental replicates. In some cases, the standard deviations are very small and cannot be visualized. Values of [C_2_mim][NTf_2_] were taken from Tomé et al. [28], [C_2_mim][C(CN)_3_] from Tomé et al. [39], [C_2_mim][TFSAM] from Gouveia et al. [44], and [C_2_mim][BF_4_] from Li et al. [58].

**Figure 4:**
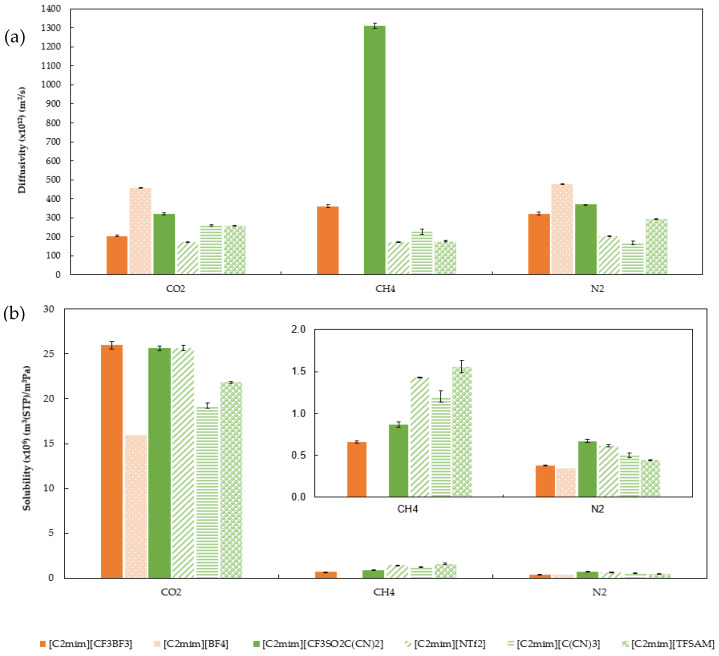
(**a**) Gas diffusivities (D) and (**b**) solubilities (S) of [C_2_mim][CF_3_BF_3_] and [C_2_mim][CF_3_SO_2_C(CN)_2_] at T = 293.15 K. In some cases, the standard deviations are very small and cannot be visualized. Values of [C_2_mim][NTf_2_] were taken from Tomé et al. [28], [C_2_mim][C(CN)_3_] taken from Tomé et al. [29], [C_2_mim][TFSAM] taken from Gouveia et al. [44], and [C_2_mim][BF_4_] taken from Li et al. [58].

The permeabilities (P) and diffusivities (D) of CO_2_, CH_4_, and N_2_ through [C_2_mim][CF_3_BF_3_] and [C_2_mim][CF_3_SO_2_C(CN)_2_] in SILM configuration using the time lag method, at 293.15 K and 1 bar of feed pressure, are shown in Figure 3 and Figure 4. Using Equation (1), it was also possible to obtain the solubilities (S) of the mentioned three gases, which are also depicted in Figure 4. The values of the gas permeabilities, diffusivities, and solubilities at 293.15 K and 1 bar of feed pressure are presented in Table 4, whereas for 308.15 K and 1 bar of feed pressure are listed in Table S5 in Supporting Information. 

For comparison purposes, the permeation properties of other ILs with the same cation and different anions bearing the same moieties, such as [C_2_mim][NTf_2_], [C_2_mim][TFSAM], [C_2_mim][BF_4_], and [C_2_mim][C(CN)_3_], were also introduced in Figure 3 and Figure 4. 

The common gas permeability trend for ILs, consisted in the following order: P_CO2_ >> P_CH4_ > P_N2_ and reported previously [44] was found to be fair for ILs studied in this work as well (Figure 3). The new SILMs demonstrated high performance in terms of carbon dioxide permeability as compared to other SILMs given for comparison (Figure 3). The obtained results show outstanding performance of the new SILM filled with [C_2_mim][CF_3_SO_2_C(CN)_2_], for which the highest CO_2_ permeability value (1095 Barrer) at 293.15 K was measured. This value is much higher than those obtained for [C_2_mim][NTf_2_] (589 Barrer) [28] and [C_2_mim][C(CN)_3_] (667 Barrer) [29] and [C_2_mim][TFSAM] (753 Barrer) [44] at the same temperature. For [C_2_mim][CF_3_BF_3_], the CO_2_ permeability of 710 Barrer was achieved, that was found to be slightly lower than that of [C_2_mim][BF_4_] (968 Barrer) [58], meaning that the introduction of the CF_3_ group in the anion decreases gas permeability. Despite the fact that new SILMs follow the trend for gas permeabilities mentioned before, the CH_4_ permeability of [C_2_mim][CF_3_SO_2_C(CN)_2_] (152 Barrer) was much higher than that obtained for [C_2_mim][NTf_2_] (32.5 Barrer) [28] and [C_2_mim][TFSAM] (36.2 Barrer) [44]. 

The gas diffusivity and solubility through [C_2_mim][CF_3_BF_3_] and [C_2_mim][CF_3_SO_2_C(CN)_2_] is presented in Figure 4. 

For both new SILMs, the gas diffusivity followed the following gas order: CO_2 >_ N_2 >_ CH_4_, which is in agreement with the gas kinetic diameters, CO_2_ (3.30Å) _<_ N_2_ (3.64Å) _<_ CH_4_ (3.80Å)[59]. It can also be observed that [C_2_mim][CF_3_SO_2_C(CN)_2_] presents a higher diffusivity for all gases when compared to [C_2_mim][CF_3_BF_3_], following the same trend observed for gas permeability (Figure 4). Surprisingly, CH_4_ diffusivity for [C_2_mim][CF_3_SO_2_C(CN)_2_] filled SILM (1313 × 10^−12^ m^2^·s^−1^) was found to be the highest among studied SILMs and was significantly higher than for the other gases. It should be stated, that the standard deviation obtained from the three independent measurements was very small, indicating the accuracy of the mentioned result. It can also be observed that, for the three studied gases, SILM with [C_2_mim][CF_3_BF_3_] showed smaller gas diffusivity than with [C_2_mim][BF_4_], while for SILM with [C_2_mim][CF_3_SO_2_C(CN)_2_] the opposite behavior was found since the gas diffusivity is higher than that of SILMs with [C_2_mim][TFSAM], [C_2_mim][NTf_2_], and [C_2_mim][C(CN)_3_]. Although it was previously stated that there is an inverse proportionality relationship between viscosity and gas diffusivity [31,32], this cannot be used to explain the lower gas diffusivity of [C_2_mim][CF_3_BF_3_] SILM when compared to SILM filled with [C_2_mim][BF_4_], since the viscosity of the respective ILs is 30.345 mPa·s and 23.350 mPa·s (at 293.15 K), correspondingly. In addition, the high gas diffusivities of [C_2_mim][CF_3_SO_2_C(CN)_2_] SILM do not corroborate this relationship, since its viscosity (29.473 mPa·s) was higher than other comparative SILMs (16.624 mPa·s for [C_2_mim][C(CN)_3_] and 23.700 mPa·s for [C_2_mim][TFSAM]), which show lower gas diffusivities. Thus, viscosity is not the determinant factor governing gas diffusivity for the two SILMs under study.

The gas solubility data for SILMs filled with [C_2_mim][CF_3_SO_2_C(CN)_2_] and [C_2_mim][CF_3_BF_3_] are shown on Figure 4. The following trend for the solubility of gases was observed: CO_2_ >> CH_4_ > N_2_, which was found to correlate with the dependence noticed above for gas permeabilities. This indicates that the gas permeability in SILMs is governed by gas solubility [31,32,60]. It is interesting to observe that despite their quite different chemical structures, both ILs under study show a similar value for CO_2_ solubility (26.0 and 25.7 × 10^−6^ m^3^ (STP) m^−3^·Pa^−1^ for [C_2_mim][CF_3_BF_3_] and [C_2_mim][CF_3_SO_2_C(CN)_2_]), which is in agreement with the CO_2_ solubility for the rest of ILs under comparison, except for [C_2_mim][BF_4_] that presents lower solubility (15.9 × 10^−6^ m^3^ (STP) m^−3^·Pa^−1^). The low CO_2_ solubility for [C_2_mim][BF_4_] can be attributed to the smaller molar volume presented in Table S2. As for CH_4_ and N_2_ solubilities, their values are one order of magnitude smaller, namely 0.70 × 10^−6^ and 0.38 × 10^−6^ m^3^ (STP) m^−3^·Pa^−1^ for [C_2_mim][CF_3_BF_3_], whereas for [C_2_mim][CF_3_SO_2_C(CN)_2_], these gas solubilities show twice higher values, 0.9 × 10^−6^ and 0.7 × 10^−6^ m^3^ (STP) m^−3^·Pa^−1^. No correlation was found between the anion size and CO_2_ permeability for both ILs [C_2_mim][CF_3_BF_3_] and [C_2_mim][CF_3_SO_2_C(CN)_2_], taking into account that the cation is the same for both Ils. However, when CO_2_ solubility was considered, an almost linear relationship could be obtained, as also found for other ILs with similar cation [33]. Although it is not possible to directly compare the CH_4_ solubility in [C_2_mim][CF_3_BF_3_] with that in [C_2_mim][BF_4_], one can see it is the lowest among all ILs selected for comparison. [C_2_mim][CF_3_SO_2_C(CN)_2_] presents the lowest CH_4_ solubility value of the related ILs, although the opposite behavior occurs for N_2_ solubility. 

### 2.5. Temperature Effect on Permeation Properties

**Figure 5:**
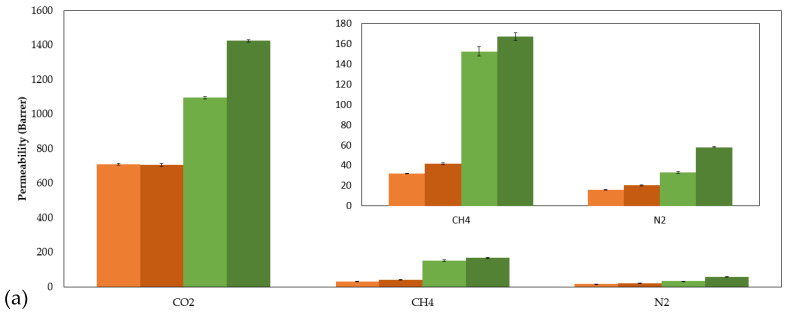

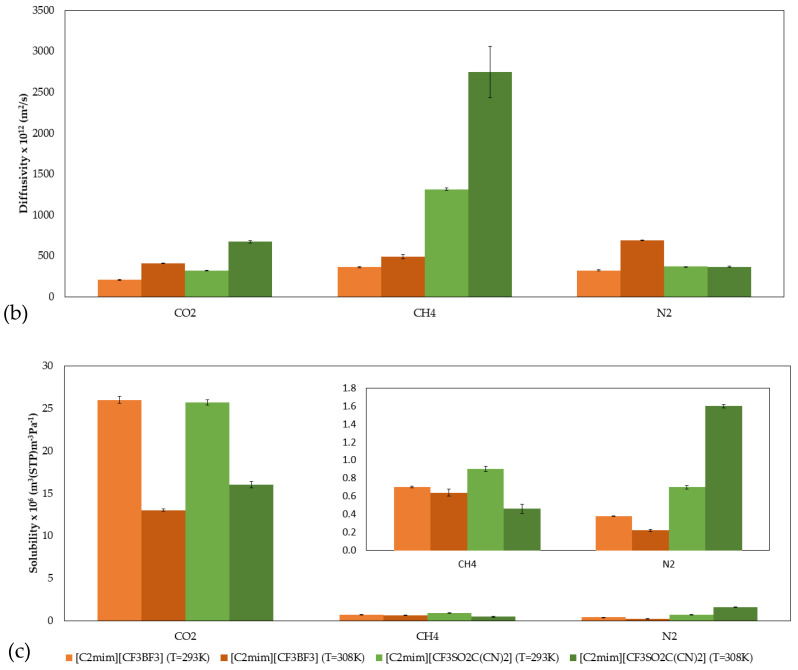
Comparison of (**a**) gas permeability, (**b**) diffusivity (D), and (**c**) solubility (S) of CO_2_, N_2_, and CH_4_ for [C_2_mim][CF_3_BF_3_] and [C_2_mim][CF_3_SO_2_C(CN)_2_] at 293.15 K and 308.15 K.

It is well known that temperature has a direct and visible effect in gas permeation properties through ILs [61]. The effect of temperature was also here studied for [C_2_mim][CF_3_BF_3_] and [C_2_mim][CF_3_SO_2_C(CN)_2_] filled SILMs. The values for gas permeability, diffusivity, and solubility at 308.15 K, for the three studied gases, are listed in Table S5 and compared with those obtained at 293.15 K in Figure 5.

As expected, Figure 5a shows that an increase in temperature generally leads to high gas permeability for all the gases for the two studied ILs. There is only one exception for CO_2_ permeability through [C_2_mim][CF_3_BF_3_] SILM, where no significant change was observed, since very similar values were found for both temperatures: 710 and 706 Barrer at 293.15 and 308 K, respectively. For the other two gases, an increase of 30% and 27% for CH_4_ and N_2_ permeabilities was obtained when comparing the two temperatures. Concerning [C_2_mim][CF_3_SO_2_C(CN)_2_], all the different gas permeabilities increased with increasing temperature. Specifically, the CO_2_ permeability changed from 1095 to 1424 Barrer, showing a rise of 30%. The greatest gas permeability increase (>75%) was reached for N_2_gas, whereas for CH_4_ only a rise of 10% was obtained.

This permeability behavior can be linked to gas diffusivity results shown in Figure 5b, that were almost doubled at 308.1K. For the CO_2_ diffusivity, both membranes filled with [C_2_mim][CF_3_BF_3_] and [C_2_mim][CF_3_SO_2_C(CN)_2_] showed an increase of 99 and 110%, while for CH_4_ diffusivity, an increase in 36 and 109% were achieved. The N_2_ diffusivities presented an increase of 115% for [C_2_mim][CF_3_BF_3_] SILM, but at the same time no significant temperature effect was observed for [C_2_mim][CF_3_SO_2_C(CN)_2_] SILM. Regarding gas solubilities depicted in Figure 5c, this parameter tends generally to decrease with increase in temperature. An average decrease of 37% was obtained for both SILMs independently of the nature of the gas under study. The exception was noticed for N_2_ solubility in [C_2_mim][CF_3_SO_2_C(CN)_2_], where an increase of 129% was indicated with the increase in temperature.

In conclusion, the increase in gas permeability with temperature can be ascribed to the enhanced molecular diffusion through the SILMs, according to the solution-diffusion model [62]. As the temperature increases, the diffusion of gas molecules through the SILM increases due not only to the higher gas kinetic energy, but also because of the decrease in the viscosity of ILs [63]. 

### 2.6. CO_2_ Separation Performance

The CO_2_/N_2_ and CO_2_/CH_4_ ideal permselectivities of the studied SILMs at temperatures of 293.15 K and 308.15 K are summarized in Table 5.

As expected from the single gas permeabilities discussed before for the three studied gases through the new SILMs, a higher ideal permselectivity was obtained for the CO_2_/N_2_ gas pair in comparison to CO_2_/CH_4_. This behavior is generally observed for ILs [64]. Comparing the permselectivity results for [C_2_mim][CF_3_BF_3_] and [C_2_mim][CF_3_SO_2_C(CN)_2_], the [C_2_mim][CF_3_BF_3_] SILM presents higher permselectivity independently of the gas pair in comparison with [C_2_mim][CF_3_SO_2_C(CN)_2_] SILM.

It can also be observed that the permselectivity of [C_2_mim][CF_3_BF_3_] and [C_2_mim][CF_3_SO_2_C(CN)_2_] SILMs decreased with the increase in temperature for both CO_2_/N_2_ and CO_2_/CH_4_ gas pairs. Specifically, the permselectivity of [C_2_mim][CF_3_BF_3_] for both gas pairs showed a decrease of 24% and 22%, respectively. For [C_2_mim][CF_3_SO_2_C(CN)_2_] SILM with an increase in temperature, a 25% decrease for CO_2_/N_2_ and an 18% increase for CO_2_/CH_4_ permselectivities was observed. The CO_2_/N_2_ permselectivity decrease with temperature stems from the solubility selectivity and, in fact, gas solubility decreases with the rise in temperature [65]. 

### 2.7. Membranes Performance Comparison

**Figure 6:**
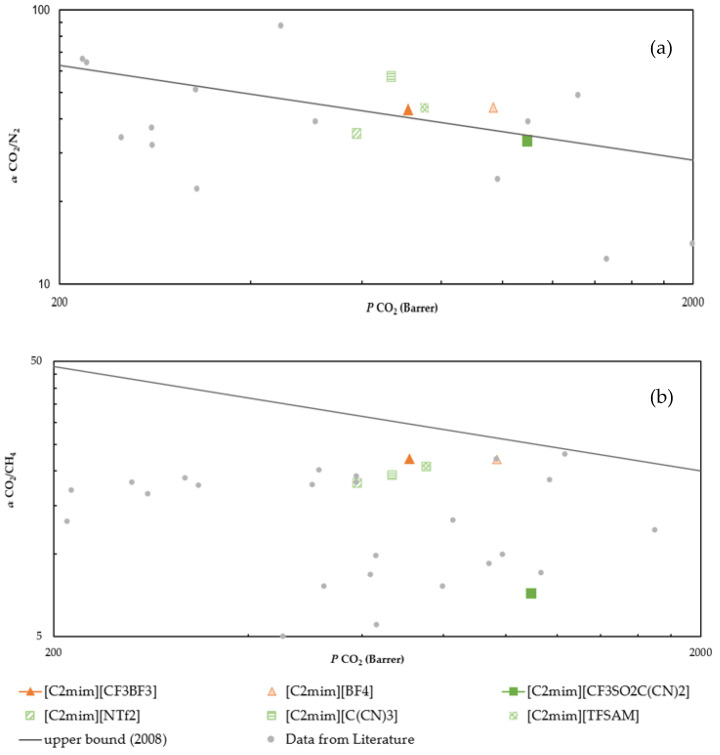
CO_2_/N_2_ (**a**) and CO_2_/CH_4_ (**b**) separation performance of [C_2_mim][CF_3_BF_3_] and [C_2_mim][CF_3_SO_2_C(CN)_2_] SILMs Robeson plots [45]. Literature data for other SILMs was used for comparison [28,29,57,64,66,67,68,69,70,71,72,73].

To better interpret the permeability together with the ideal permselectivity results for CO_2_/N_2_ and CO_2_/CH_4_ pair of gases, the Robeson plots, presented in Figure 6a,b, were applied. The Robeson plot upper limit for each gas pair represents an empirical limit proposed in 2008 by Robeson, through the correlation of a substantially amount of gas permeability and selectivity data. The area in the right hand corner corresponding to high CO_2_ permeability and simultaneously high CO_2_/N_2_ or CO_2_/CH_4_ selectivity is the target region, where in 2008 no data were yet available.

It can be seen from Figure 6a that [C_2_mim][BF_4_] and [C_2_mim][CF_3_BF_3_] have similar CO_2_/N_2_ permselectivities, but very different CO_2_ permeabilities, where the [C_2_mim][CF_3_BF_3_] shows lower CO_2_ permeability than [C_2_mim][BF_4_]. Thus, the introduction of CF_3_ group in [BF_4_]^-^ anion does not represent a step forward either in terms of CO_2_ permeability or CO_2_/N_2_ selectivity. In addition, to be noted that [C_2_mim][CF_3_BF_3_] performance is very similar to that of [C_2_mim][TFSAM], despite the very different chemical structures of the anions. As for [C_2_mim][CF_3_SO_2_C(CN)_2_] IL, its CO_2_/N_2_ permselectivity is similar to that [C_2_mim][NTf_2_] but smaller than that of [C_2_mim][TFSAM] and [C_2_mim][C(CN)_3_], meaning than the enhancement of selectivity gained from [C_2_mim][NTf_2_] to [C_2_mim][TFSAM] is here lost. However, a significant gain in terms of CO_2_ permeability was achieved by the new SILM filled with [C_2_mim][CF_3_SO_2_C(CN)_2_]. Overall, this SILM is on top of the Robeson plot line, meaning that it shows a good selectivity when compared to the state-of-the-art membranes, and demonstrates an improvement in terms of CO_2_ permeation when compared of other ILs, representing a step forward to overcome the low gas flux of IL-based membranes. 

In what concerns the CO_2_/CH_4_ separation, and as discussed earlier, the fact that [C_2_mim][CF_3_SO_2_C(CN)_2_] has a higher CH_4_ diffusivity, and consequently higher permeability, than those SILMs used for comparison, greatly affects CO_2_/CH_4_ selectivity. So, despite the great increase in CO_2_ permeability, its permselectivity suffers a great reduction. For example, comparing SILMs permselectivity filled with [C_2_mim][CF_3_SO_2_C(CN)_2_] to that of [C_2_mim][NTf_2_] and [C_2_mim][TFSAM] SILMs, 60% and 65% loss in selectivity was observed, respectively. However, [C_2_mim][CF_3_SO_2_C(CN)_2_] shows higher CO_2_ permeability when compared to the same two ILs, presenting an increase of 86% and 45%, respectively. A direct comparison of [C_2_mim][CF_3_BF_3_] with [C_2_mim][BF_4_] shows a decrease in CO_2_ permeability of 27%, maintaining approximately the same selectivity (22.2 for [C_2_mim][CF_3_BF_3_] and 22.0 for [C_2_mim][BF_4_]).

## 3. Materials and Methods

### 3.1. Materials

Acetonitrile (MeCN, anhydrous 99.9%, Acros, Waltham, MA, USA), dichloromethane (DCM, 99.6%, Acros), isopropyl alcohol (*i*-PrOH, 99.7%, Sigma Aldrich, St. Louis, MO, USA), 1-ethyl-3-methyl-imidazolium bromide ([C2mim][Br], 98%, Iolitec, Heilbronn, Germany), lithium hydride (95%, Sigma Aldrich), trimethyl borate (B(OMe)_3_, 98.0%, Sigma Aldrich), (trifluoromethyl)trimethylsilane (99%, Sigma Aldrich), boron trifluoride diethyl etherate (BF_3_·Et_2_O, Sigma Aldrich), and trifluoromethanesulphonyl fluoride (CF_3_SO_2_F, 98% ABCR, Karlsruhe, Germany) were used without further purification. Carbon dioxide (CO_2_), nitrogen (N_2_), and methane (CH_4_) were all supplied by Air Liquide and were of, at least, 99.99% purity. Gases were used as received without further purification. Tetrahydrofuran (THF) was purified by refluxing over the deep purple sodium-benzophenone complex. Propionitrile (EtCN, 99%, Sigma Aldrich) was distilled over CaH_2_. Triethylamine (99.5%, Merck, Darmstadt, Germany) was distilled under inert atmosphere over metal Na. Malononitrile (99%, Sigma Aldrich) was distilled under reduced pressure. Potassium fluoride (KF, 99.97%, Sigma Aldrich) was dried in a stainless-steel pan using a hot plate (Severin, Sundern, Germany, 1500 W) set to maximum heating (T > 200 °C) for 2–3 h with stirring. After cooling to ~70 °C, it was quickly transferred to the heat-treated glass flask, then stored under inert atmosphere. Anhydrous magnesium sulfate was prepared in-house from MgSO4·7H_2_O (MgSO_4_, >99%, Sigma Aldrich) by heating a saturated aqueous solution of magnesium sulfate in a stainless-steel pan (~500 mL) using a hot plate (Severin 1500 W) set to maximum heating. Following evaporation of all liquid water, heating was continued for another ~6–8 h (T > 200 °C) to obtain an anhydrous cake. The cake was allowed to cool to ~70 °C, broken into ~1-cm sized pieces, then stored in a sealed glass jar prior to use. Porous hydrophobic poly(vinylidene fluoride) (PVDF) Durapore^®^ supports, with a pore size of 0.22 μm and average thickness of 125 μm, were provided by the Merck Millipore Corporation.

### 3.2. Synthesis

#### 3.2.1. Potassium Trifluoro(Trifluoromethyl)Borate (KCF_3_BF_3_)

The 150 mL of anhydrous THF, freshly dried KF (10.00 g, 172.3 mmol), trimethyl borate (19.68 g, 189.3 mmol), and (trifluoromethyl)trimethylsilane (26.90 g, 189.3 mmol) were added to the four-necked round bottom flask equipped with a reflux condenser, thermometer, mechanical stirrer, and dropping funnel under inert atmosphere. The suspension was slowly heated to 50 °C and the reaction was continued for 5–6 h at 50 °C until the formation of a clear transparent solution. The solution was further cooled down to –25 °C and after the dropwise addition of BF_3_·Et_2_O (21.35 g, 150.5 mmol), the reaction was allowed to warm up to RT. The stirring was continued at RT for 3 h, whereupon the reflux condenser was replaced by a distillation system and 50 mL of THF were distilled off. The 50 mL of *i*-PrOH were added to the mixture and the THF residue was evaporated until the vapor temperature reached 100 °C. The reaction mass was cooled down to RT, the precipitate was filtered, collected, and dried at 25 °C/0.1 mbar for 2 h. Yield: 19.9 g (60%); *T*_m_ > 350 °C; ^13^C NMR (150.9 MHz, DMSO-d_6_), δ (ppm): 131.2 (m); ^19^F NMR (564.7 MHz, DMSO-d_6_), δ (ppm): −73.6 (dd, J = 64.8, 32.1 Hz, CF_3_), −153.4 (m, F); ^11^B NMR (192.6 MHz, DMSO-d_6_), δ (ppm): −1.43 (qq, J = 39.8, 32.5 Hz); and IR (ATR-mode), cm^−1^: 1191 (w), 1031 (vs, ν_BF_), 982 (s), 944 (vs, ν_CF_), 730 (m), 653 (s, ν_BF_), 623 (w).

#### 3.2.2. 1-Ethyl-3-Methyl-Imidazolium Trifluoro(Trifluoromethyl)Borate [C2mim][CF_3_BF_3_]

The 1-ethyl-3-methyl-imidazolium bromide (16.95 g, 88.7 mmol) was quickly charged in the Schlenk flask. The flask was evacuated on vacuum at RT for 15 min and filled with inert atmosphere (Ar). The 30 mL of anhydrous acetonitrile were injected via syringe and stirring was continued at RT until the formation of a clear solution. Further on, the solution of potassium trifluoro(trifluoromethyl)borate (15.61 g, 97.6 mmol) in 30 mL of anhydrous acetonitrile was added dropwise via syringe at RT. The formation of the milky suspension was observed immediately. The resultant suspension was stirred overnight at RT, whereupon the precipitated KBr was filtered off and acetonitrile was evaporated under reduced pressure. The crude product was dissolved in 200 mL of DCM, washed with H_2_O (4 × 40 mL), and dried over anhydrous magnesium sulfate. MgSO_4_ was filtered off and decolorizing carbon black was added to the light-yellow solution. The suspension was stirred at RT for 2 h and carbon black was filtered off, the dichloromethane was evaporated under reduced pressure and the resultant colorless transparent oil was dried at 55 °C/0.1 mbar for 1 d with a special flask filled with P_2_O_5_ and introduced into the vacuum line. Yield: 16.5 g (75%); *T*_cr_ = −71.9 °C, *T*_m_ = −17.9 °C (DSC); *T*_onset_ = 215 °C (TGA); η = 25.592 mPa·s or cP (at 25 °C); ρ = 1.3382 g·cm^−3^ (at 25 °C); ^1^H NMR (600.2 MHz, DMSO-d_6_), δ (ppm): 9.05 (s, 1H), 7.72 (s, 1H), 7.64 (s, 1H), 4.19 (q, J = 7.3 Hz, 2H), 3.84 (s, 3H), 1.42 (t, J = 7.3 Hz, 3H); ^13^C NMR (150.9 MHz, DMSO-d_6_), δ (ppm): 136.3, 123.6, 122.0, 44.2, 40.0, 35.6, 14.9; ^19^F NMR (564.7 MHz, DMSO-d_6_), δ (ppm): −153.7 (m, F), −73.8 (m, CF_3_); ^11^B NMR (192.6 MHz, DMSO-d_6_), δ (ppm): −1.44 (qq, J = 39.8, 32.5 Hz); IR (ATR-mode), cm^−1^: 3171 (m, ν_CH_), 3128 (m, ν_CH_), 2990 (w, ν_CH_), 1573 (m), 1462 (w), 1432 (w), 1392 (w), 1335 (w), 1171 (s), 1057 (vs, ν_BF_), 977 (m), 952 (vs, ν_CF_), 841 (w), 750 (w), 634 (s, ν_BF_); Calc. for C_7_H_11_BF_6_N_2_ (248.09): C, 33.90%; H, 4.47%; F, 45.97%; and Found: C, 34.02%; H, 4.58%; F, 45.86%.

#### 3.2.3. Triethylammonium Dicyano((Trifluoromethyl)Sulfonyl)Methanide [NC_2_C_2_C_2_H][CF_3_SO_2_C(CN)_2_] 

Malononitrile (4.07 g, 61.6 mmol) and freshly distilled triethylamine (13.72 g, 135.6 mmol) were dissolved in 60 mL of anhydrous EtCN under inert atmosphere at RT. The reaction solution was cooled down to −40 °C in the cold bath (CCl_4_ + dry ice). The trifluoromethanesulphonyl fluoride (10.31 g, 67.8 mmol) was distilled in the pre-weighted thick-walled glass trap from the balloon and then redistilled in the dropping funnel containing 15 mL of anhydrous EtCN and equipped with a cooling jacket precooled to −78 °C. The dropping funnel with the CF_3_SO_2_F solution was attached to the reaction flask and was added dropwise under inert atmosphere. The reaction mixture was stirred at −40 °C for 1 h and then allowed to warm up to RT. Stirring was continued at RT for an additional 12 h, whereupon the solvent was removed under reduced pressure at 35 °C/10 mbar and the obtained brown oil was dried at 35 °C/0.1 mbar for 3 h. The residual brownish oil was dissolved in 90 mL of DCM and washed with water (4 × 90 mL). The organic layer was dried over anhydrous MgSO_4_, which was further filtered off and the solvent was removed at reduce pressure. The product in a form of light-brown oil was dried at 55 °C/0.1 mbar for 5 h with a special flask filled with P_2_O_5_ and introduced into the vacuum line. Yield: 15.8 g (86%); ^1^H NMR (400.1 MHz, DMSO-d_6_), δ (ppm): 8.96 (br. s., 1H), 3.12 (s, 6H), 1.20 (s, 9H); ^13^C NMR (100.6 MHz, DMSO-d_6_), δ (ppm): 126.9–116.2 (q, J_CF_ = 321 Hz, CF_3_), 116.0, 46.2, 36.2, 8.8; ^19^F NMR (376.5 MHz, DMSO-d_6_), δ (ppm): −81.8 (m, CF_3_).

#### 3.2.4. Lithium Dicyano((Trifluoromethyl)Sulfonyl)Methanide (Li CF_3_SO_2_C(CN)_2_)

The suspension of LiH (0.86 g, 107.6 mmol) in 20 mL of anhydrous THF was slowly added to the solution of triethylammonium dicyano((trifluoromethyl)sulfonyl)methanide (10.74 g, 35.9 mmol) in 50 mL of anhydrous THF preliminary cooled down to 0 °C under inert atmosphere (Ar). Caution: Immediately after the start of the reaction, gas evolution was observed. The reaction mixture was warmed up to room temperature and stirred at 35 °C for 2 h. The excess of LiH was filtered off, the solvent was removed at reduced pressure, and the residue was dried at 60 °C/0.1 mbar for 3 h. The anhydrous CH_2_Cl_2_ was added to the crude product representing viscous brown oil and the mass was stirred mechanically under the inert flow till the formation of the yellow powder. The powder was collected by filtration and dried at 60 °C/10 mbar for 2 h, whereupon it was dissolved in 90 mL of anhydrous CH_3_CN and refluxed with carbon black for 2 h. The charcoal was filtered off, the solvent was removed under reduced pressure, and the light-yellow viscous oil was dried at 60 °C/10 mbar for 2 h. The anhydrous DCM was added to the oil and the mass was stirred mechanically under the inert flow till the formation of the pale-yellow powder. The powder was collected by filtration and dried at 60 °C/0.1 mbar for 12 h. Yield: 6.31 g (86%); *T*_m_ = no melting point determined (decomposition started >260 °C); ^13^C NMR (100.6 MHz, DMSO-d6), δ (ppm): 139.1, 136.5, 126.6–116.9 (q, J_CF_ = 321 Hz, CF_3_), 116.9, 116.5; ^19^F NMR (376.5 MHz, DMSO-d_6_), δ (ppm): −81.8 (s, CF_3_); ^7^Li NMR (155.5 MHz, DMSO-d_6_), δ (ppm): −1.01 (s); IR (KBr), cm^−1^: 2234 (s, ν_CN_), 2214 (vs, ν_CN_), 2197 (vs, ν_CN_), 1352 (vs, ν_asSO_), 1218 (vs, ν_CF_), 1185 (vs), 1155 (s, ν_sSO_), 1080 (m, ν_CF_), 765 (w), 675 (s), 607 (m), 588 (s); Calc. for C_4_F_3_N_2_O_2_SLi (204.05): C, 23.54%; N, 13.73%; F, 27.93%; and Found: C, 23.43%; N, 13.98%; F, 27.83%.

#### 3.2.5. 1-Ethyl-3-Methyl-Imidazolium Dicyano((Trifluoromethyl)Sulfonyl)Methanide [C_2_mim][CF_3_SO_2_C(CN)_2_]

The solution of lithium dicyano((trifluoromethyl)sulfonyl)methanide (6.00 g, 29.4 mmol) in 10 mL of H_2_O was added dropwise to the solution of 1-ethyl-3-methyl-imidazol-ium bromide (5.35 g, 28.0 mmol) in 10 mL of H_2_O at RT under vigorous stirring. The formation of an emulsion was observed immediately and the stirring was continued for 2 h at RT. Organic oil was extracted with 80 mL of DCM. Organic layer was washed with H_2_O (4 × 10 mL) and dried over anhydrous magnesium sulfate. MgSO_4_ was filtered off, the dichloromethane was evaporated at 50 °C under reduced pressure, and resultant brown oil was dried at 55 °C/0.1 mbar for 1 day. Yield: 7.91 g (92%); *T*_g_ = −84.1 °C (DSC); *T*_onset_ = 300 °C (TGA); η = 25.003 mPa·s or cP (at 25 °C); η = 26 cP (at 25 °C); ρ = 1.3288 g·cm^−3^; ^1^H NMR (400 MHz, DMSO-d_6_), δ (ppm): 9.15 (s, 1H), 7.76 (s, 1H), 7.67 (s, 1H), 4.22 (q, J = 7.3 Hz, 2H), 3.88 (s, 3H), 1.45 (t, J = 7.3 Hz, 3H); ^13^C NMR (400 MHz, DMSO-d_6_), δ (ppm): 136.5, 125.6–115.9 (q, J_CF_ = 321 Hz, CF_3_), 123.7, 122.1, 115.5, 44.3, 35.7, 34.8, 14.9; ^19^F NMR (400 MHz, DMSO-d_6_), δ (ppm): −81.5 (s, CF_3_); IR (KBr), cm^−1^: 3158 (s, νCH), 3119 (s, νCH), 2990(m, νCH), 2505 (w), 2414 (w), 2203 (s, ν_CN_), 2186 (vs, ν_CN_), 2143 (w, ν_CN_) 1572 (s), 1456 (m), 1350 (vs, ν_asSO_), 1209 (vs, ν_CF_), 1181 (vs), 1114 (m, ν_sSO_), 1071 (s, ν_CF_), 960 (w), 842 (m), 756 (m), 701 (w), 662 (vs), 623 (m), 583 (s), 535 (w); Calc. for C_10_H_11_F_3_N_4_O_2_S (308.28): C, 38.96%; H, 3.60%; N, 18.17%; F, 18.49%; and Found: C, 39.15%; H, 3.70%; N, 18.01%; F, 18.42%. 

### 3.3. Analytical and Physicochemical Measurements

#### 3.3.1. Water Content

In order to reduce the content of water and other volatile substances, all IL samples were additionally dried at approximately 1 Pa and 318 K for at least 4 days and their H_2_O content was determined by Karl Fischer titration using an 831 KF Coulometer (Metrohm).

#### 3.3.2. Spectroscopical Properties

NMR spectra were recorded on AMX-400 and AMX-600 spectrometers (Bruker, Germany) at 25 °C in the indicated deuterated solvent and are listed in ppm. The signal corresponding to the residual protons of the deuterated solvent was used as an internal standard for ^1^H and ^13^C NMR, while for ^19^F NMR, the C_6_F_6_ (−162.5 ppm) was utilized as an external standard. IR spectra were acquired on a Magna-750 (Nicolet Instrument Corporation) or on Tensor 27 (Brucker, Germany) Fourier IR-spectrometer using ATR technology (128 scans, resolution is 2 cm^−1^) and Spectragryph optical spectroscopy software [74]. 

#### 3.3.3. Thermal Properties

Thermal gravimetric analysis (TGA) was carried out in air on a TGA2 STARe System (Mettler Toledo, Switzerland) applying a heating rate of 5 °C min^−1^. The onset weight loss temperature (*T*_onset_) was determined as the point in the TGA curve at which a significant deviation from the horizontal was observed. The resulting temperature was then rounded to the nearest 5 °C. For Differential Scanning Calorimetry (DSC) measurements, all samples were hermetically sealed in Al pans inside the argon-filled glove-box (MBRAUN MB-Labstar, H_2_O and O_2_ content < 0.5 ppm). DSC of [C2mim][CF_3_BF_3_] and [C2mim][CF_3_SO_2_C(CN)_2_] samples was performed on a DSC3+ STARe System (Mettler Toledo, Switzerland) differential calorimeter applying a heating rate of 2 °C min^−1^ in the range of −90 to 150 °C. The glass transition (*T*_g_) and melting (*T*_m_) temperatures were determined during second heating cycle. Crystallization temperature (*T*_cr_) was determined during second cooling cycle. For simplicity and convenience, the temperatures of thermal transitions were measured as maxima of the peaks corresponding to the endothermic or exotermic heat effects.

#### 3.3.4. Thermophysical Properties

Measurements of viscosity (η) and density (ρ) were carried out in the temperature range from 293.15 K up to 353.15 K and at atmospheric pressure using an Anton Paar (model SVM 3000) automated rotational Stabinger viscometer-densimeter, with a temperature uncertainty of ±0.01 K. The relative uncertainty of the dynamic viscosity is ±0.25%, and the absolute uncertainty of the density is ±0.0005 g·cm^−^^3^. Triplicate measurements were carried out and standard deviations were calculated. 

#### 3.3.5. Supported Ionic Liquid Membranes (SILMs) Preparation

The studied ILs were supported on porous hydrophobic PVDF supports using vacuum, as previously described [30,75]. In short, the porous membrane filter was firstly placed inside a vacuum chamber for about 1 h in order to remove any impurities and/or the air within the pores. Afterwards, while keeping the vacuum in the chamber, a few drops of the studied ILs were carefully placed on the membrane surface. To ensure proper impregnation, the SILM was left for over an hour inside the vacuum chamber, after which it was taken out and the IL’s excess was wiped with paper tissue. 

#### 3.3.6. Gas Permeation Experiments

Ideal CO_2_ and N_2_ permeabilities and diffusivities through the prepared membranes were measured at 293.15 K and 308.15 K using an apparatus with a time-lag method implemented, whose detailed description was previously reported [76]. The SILMs were inserted into the permeation cell and degassed under vacuum (<0.1 kPa) for 12 h. Then, gas permeation experiments were conducted in such a way that three CO_2_ and N_2_ gas independent measurements were carried out for each membrane sample. In between each run, the permeation cell and lines were thoroughly vacuumed.

The solution-diffusion mass transport model was used to describe the gas transport through the prepared SILMs. Consequently, after measuring the permeability (P) and the diffusivity (D), the solubility (S) can be calculated using Equation (1).

(5) P=D×S

A critical parameter to quantify membrane performance is the ideal permselectivity, $\alpha_{i,j}$, that can be calculated as shown in Equation (2):

(6) $$\alpha_{i,j}=\frac{P_{i}}{P_{j}}=\left(\frac{D_{i}}{D_{j}}\right)\times \left(\frac{S_{i}}{S_{j}}\right)$$

where *i* is the most permeable gas and *j* is the less permeable gas.

## 4. Conclusions

In the present study, two ILs containing common cation ([C_2_mim]^+^) and two asymmetric anions, namely [C_2_mim][CF_3_SO_2_C(CN)_2_] and [C_2_mim][CF_3_BF_3_], were successfully prepared. The alkali dicyano((trifluoromethyl)sulfonyl)methanide salt and the [C_2_mim][CF_3_SO_2_C(CN)_2_] IL were here synthesized for the first time. The density and viscosity of both ILs were measured in the temperature range between 293.15 K and 353.15 K and atmospheric pressure and compared to those properties of ILs bearing structurally similar symmetric anions. Finally, two new ILs were further used as liquid phases for the formation of SILMs and their CO_2_/N_2_ and CO_2_/CH_4_ gas separation properties were studied in detailed.

The application of “asymmetric principle” for the design of new anions led to the mixed results that were found to be dependent on the type of the functional group introduced in the anion. The substitution of one fluorine atom with the CF_3_ group in the BF_4_ anion resulted in a pronounced decrease at melting point (from 15 to −17.9 °C), a reduction of thermal stability (from *T*_onset_ = 420 to 215 °C). In terms of permeation properties, a decrease in CO_2_, N_2_, and CH_4_ single gas permeabilities (from 968, 44.0 and 22.2 to 710, 32.0, 16.4 Barrer at 293.15 K, and 1 bar of feed pressure, respectively).

For simplicity and accuracy, the comparison of [C_2_mim][CF_3_SO_2_C(CN)_2_] was performed with [C_2_mim][C(CN)_3_]. Thus, the substitution of the CN group with the CF_3_SO_2_ group led to the disappearance of crystallization or melting processes, and an increase in thermal stability (from *T*_onset_ = 270 to 300 °C). In terms of permeation properties, an increase in CO_2_ and CH_4_ permeabilities (1095 and 667, 152 and 34.4 Barrer at 293.15 K, and 1 bar of feed pressure, respectively), being this last one due to a remarkable increase in the CH_4_ diffusivity.

In terms of viscosity, two different behaviors were here observed, as the introduction of CF_3_ in [C_2_mim][BF_4_] leads to an [C_2_mim][CF_3_BF_3_] IL with slightly higher viscosity than the former, probably due to the bulkiness and rigidity of the CF3 group, while [C_2_mim][CF_3_SO_2_C(CN)_2_] presents a lower viscosity than the corresponding IL with symmetrical anion structure [C_2_mim][NTf_2_]. The introduction of CN groups in the IL’s anion to achieve asymmetry has already been shown to be a valuable strategy to provide ILs with low viscosity.

Regarding the two SILMs performances at 293 K, [C_2_mim][CF_3_SO_2_C(CN)_2_] is better positioned on the Robeson plot for the separation of the (CO_2_/N_2_) gas pair than [C_2_mim][CF_3_BF_3_], with higher permeability (1095 Barrer) and considerable permselectivity (33.3). However, this picture changes when it comes to the separation of (CO_2_/CH_4_) gas pair, where [C_2_mim][CF_3_BF_3_] SILM permselectivity is considerably higher (22.2) than that of IL [C_2_mim][CF_3_SO_2_C(CN)_2_], which is 7.2, with the consequent trade-off in CO_2_ permeability (710 Barrer).

To conclude, the change from [BF_4_]^−^ to [CF_3_BF_3_]^−^ anion does not represent a step forward in the improvement of SILMs, neither in terms of CO_2_ permeability nor in CO_2_/N_2_ selectivity. The [C_2_mim][CF_3_SO_2_C(CN)_2_] SILM was found to be on top of the Robeson plot line, showing good (CO_2_/N_2_) permselectivity when compared to the state-of-the-art membranes, and demonstrating an improvement in terms of CO_2_ permeation when compared of other ILs, representing a significant advancement in overcoming the low gas flux of IL-based membranes.

## Figures and Tables

**Table 1:** Fitted parameters for the temperature dependence of density for [C_2_mim][CF_3_BF_3_] and [C_2_mim][CF_3_SO_2_C(CN)_2_].

Linear Fitting	*a* (g·cm^−3^)	*b* × 10^4^ (g·cm^−3^·K^−1^)	*c* × 10^6^ (g·cm^−3^·K^−2^)	R^2^
[C_2_mim][CF_3_BF_3_]	2.56	0.655	8.26	0.9999
[C_2_mim][CF_3_SO_2_C(CN)_2_]	1.576	−8.79	-	1.000

**Table 2:** Fitted parameters of Equation (4) for the viscosity of [C_2_mim][CF_3_BF_3_] and [C_2_mim][CF_3_SO_2_C(CN)_2_] with temperature.

Parameter Fitting	*η*_∞_ × 10^3^ (mPa·s)	*E_a_* (kJ·mol^−1^)	R^2^
[C_2_mim][CF_3_BF_3_]	6.83	20.35	0.9989
[C_2_mim][CF_3_SO_2_C(CN)_2_]	2.26	23.02	0.9965

**Table 3:** Comparison of density, viscosity, and molar volume of [C_2_mim][CF_3_BF_3_] and [C_2_mim][CF_3_SO_2_C(CN)_2_] with those of other ILs bearing anions with the same moieties at 298.15 K and atmospheric pressure.

Linear Fitting	wt% of Water	*M* (g·mol^−1^)	*η* (mPa·s) ^1^	*ρ* (g·cm^−3^) ^1^	*V_m_* (cm^3^·mol^−1^) ^2^
[C_2_mim][CF_3_BF_3_]	0.21	248.09	29.47	1.344	384.61
[C_2_mim][CF_3_SO_2_C(CN)_2_]	0.17	308.28	30.35	1.318	233.86
[C_2_mim][NTf_2_] ^3^	0.02	391.31	39.08	1.524	256.78
[C_2_mim][N(C_2_F_5_SO_2_)_2_] ^4^	0.02	491.33	85.50	1.599	307.3
[C_2_mim][N(CN)_2_] ^3^	0.09	177.21	17.95	1.106	160.24
[C_2_mim][C(CN)_3_] ^5^	0.01	201.10	16.62	1.085	185.54
[C_2_mim][TFSAM] ^6^	0.02	284.26	23.70	1.352	210.30
[C_2_mim][BF_4_] ^7^	0.03	198.09	23.35	1.287	155.90

^1^ Density (*ρ*) and viscosity (*η*) measured at 293.15 K. ^2^ Molar volume (*V*_M_) obtained at 293.15 K. ^3^ from Tomé et al. [28] ^4^, from Gouveia et al. [30] ^5^, from Tomé et al. [29] ^6^, from Gouveia et al. [44] ^7^, and from Scovazzo et al. [57].

**Table 4:** Gas permeability **^1^** (P), diffusivity (D), and solubility (S) of the [C_2_mim][CF_3_BF_3_] and [C_2_mim][CF_3_SO_2_C(CN)_2_] ILs in SILM configuration **^2^** at 308.15 K and 1 bar of feed pressure.

		*SILM Membrane*	
Permeation Property	Gases	[C_2_mim][CF_3_BF_3_]	[C_2_mim][CF_3_SO_2_C(CN)_2_]
P (barrer)	CO_2_	710 ± 5	1095 ± 6
	CH_4_	32.0 ± 0.1	152 ± 5
	N_2_	16.4 ± 0.4	32.8 ± 0.7
D × 10^12^ (m^2^/s)	CO_2_	205 ± 4	320 ± 5
	CH_4_	361 ± 7	1313 ± 14
	N_2_	322 ± 8	370 ± 1
S × 10^6^ (m^3^ (STP) m^−3^·Pa^−1^)	CO_2_	26.0 ± 0.4	25.7 ± 0.3
	CH_4_	0.66 ± 0.01	0.87 ± 0.03
	N_2_	0.38± 0.004	0.67 ± 0.02

**^1^** Barrer (1 Barrer = 10^−10^ cm^3^ (STP) cm cm^−2^ s^−1^ cmHg^−1^). **^2^** The listed uncertainties represent the standard deviations based on three independent experiments.

**Table 5:** CO_2_ ideal permselectivities (α) of the [C_2_mim][CF_3_BF_3_] and [C_2_mim][CF_3_SO_2_C(CN)_2_] ILs in SILM configuration at 293.15 K and 308.15 K.

IL	T (K)	CO_2_/CH_4_	σ ^1^	CO_2_/N_2_	σ ^1^
[C_2_mim][CF_3_BF_3_]	293.15	22.2	0.20	44.4	1.21
	308.15	16.9	0.14	37.0	0.92
[C_2_mim][CF_3_SO_2_C(CN)_2_]	293.15	7.2	0.28	33.2	0.85
	308.15	8.5	0.29	24.6	0.38

^1^ The listed uncertainties represent the standard deviations based on three independent experiments.

## Supplementary Materials

The following supporting information can be downloaded at, Table S1. Measured density, (“ρ”, g·cm^−3^), of the [C_2_mim][CF_3_BF_3_] and [C_2_mim][CF_3_SO_2_C(CN)_2_] ILs at atmospheric pressure. Table S2. Molar volumes, (VM, cm^3^·mol^−1^), of the [C_2_mim][CF_3_BF_3_] and [C_2_mim][CF_3_SO_2_C(CN)_2_] ILs at atmospheric pressure in the temperature range between 293.15 and 353.15 K. Figure S1. Temperature dependence of the molar volumes (VM) for [C_2_mim][CF_3_BF_3_] (·) and [C_2_mim][CF_3_SO_2_C(CN)_2_] (·). The errors bars are smaller than the symbols used to represent the experimental data. Table S3. Thermal expan-sion coefficients (αP) of the [C_2_mim][CF_3_BF_3_] and [C_2_mim][CF_3_SO_2_C(CN)_2_]. at atmospheric pressure. Table S4. Measured viscosity, (η, mPa·s), of the [C_2_mim][CF_3_BF_3_] and [C_2_mim][CF_3_SO_2_C(CN)_2_] ILs at atmospheric pressure. Table S5. Gas Permeabilitya (P). Diffusivi-ty (D) and Solubility (S) of the [C_2_mim][CF_3_BF_3_] and [C_2_mim][CF_3_SO_2_C(CN)_2_] ILs ILs at 308.15 K and 1 bar of feed pressure.

## Sample Availability

Samples of the compounds are not available from the authors.
