# Supplementary material for: Tunning CO2 Separation Performance of Ionic Liquids through Asymmetric Anions
Source: Molecules. 2022 Jan 9;27(2):413. doi: 10.3390/molecules27020413 (PMC8778609; doi:10.3390/molecules27020413)
Supplement: Supplementary file 1 [file molecules-27-00413-s001.zip › molecules-1537709-supplementary.pdf]

Supporting Information

Tunning Ionic Liquids-based Membranes for CO_2 Separation

Bruna F. Soares. José M. Pires and Isabel M. Marrucho*

Departamento de Engenharia Química e Centro de Química Estrutural. Instituto Superior de Lisboa. Universidade de Lisboa. Av. Rovisco Pais 1. 1049-001 Lisboa

* Corresponding author. Tel.: +351 218413385; fax: +351 218499242. E-mail address: *isabel.marrucho@tecnico.ulisboa.pt* (I. M. Marrucho).

Table S1. Measured density, (ρ, g.cm^{-3}), of the $[C_2mim][CF_3BF_3]$ and $[C_2mim][CF_3SO_2C(CN)_2]$ ILs at atmospheric pressure.

T(K)	ρ (g.cm^{-3})	
	**$[C_2mim][CF_3BF_3]$**	**$[C_2mim][CF_3SO_2C(CN)_2]$**
**293.15**	1.344	1.318
**298.15**	1.336	1.314
**303.15**	1.328	1.309
**308.15**	1.320	1.305
**313.15**	1.313	1.300
**318.15**	1.307	1.296
**323.15**	1.300	1.292
**328.15**	1.294	1.287
**333.15**	1.289	1.283
**338.15**	1.284	1.278
**343.15**	1.279	1.274
**348.15**	1.275	1.270
**353.15**	1.271	1.266

Table S2. Molar volumes, (V_M, cm^3.mol^{-1}), of the $[C_2mim][CF_3BF_3]$ and $[C_2mim][CF_3SO_2C(CN)_2]$ ILs at atmospheric pressure in the temperature range between 293.15 and 353.15 K.

T(K)	V_M (cm^3.mol^{-1})	
	**$[C_2mim][CF_3BF_3]$**	**$[C_2mim][CF_3SO_2C(CN)_2]$**
**293.15**	384.61	233.86
**298.15**	387.00	234.66
**303.15**	389.31	235.46
**308.15**	391.57	236.27
**313.15**	393.76	237.08
**318.15**	395.64	237.88
**323.15**	397.59	238.69
**328.15**	399.42	239.50
**333.15**	401.13	240.32
**338.15**	402.45	241.14
**343.15**	404.16	241.95
**348.15**	405.49	242.78
**353.15**	406.67	243.60

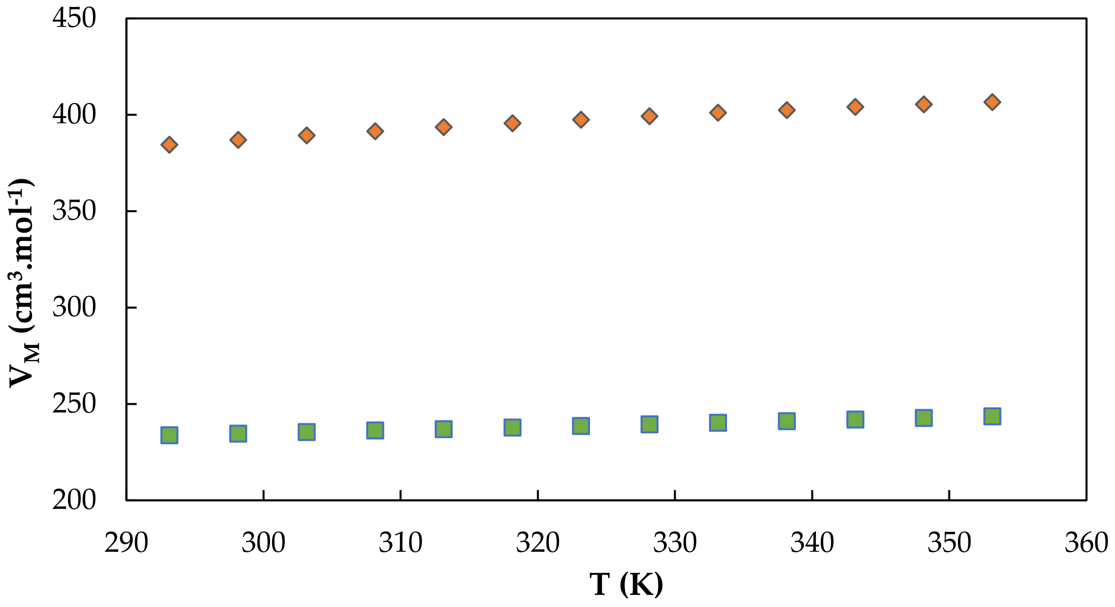

Figure S1. Temperature dependence of the molar volumes (V_M) for [C_2mim][CF_3BF_3] (◆) and [C_2mim][$CF_3SO_2C(CN)_2$] (■). The errors bars are smaller than the symbols used to represent the experimental data.

Table S3. Thermal expansion coefficients (α_P) of the [C_2mim][CF_3BF_3] and [C_2mim][$CF_3SO_2C(CN)_2$]. at atmospheric pressure.

**T(K)**	**α_P x 10^4 (K^{-1})**	
	**[C_2mim][CF_3BF_3]**	**[C_2mim][$CF_3SO_2C(CN)_2$]**
**293.15**	9.06	6.67
**298.15**	9.10	6.69
**303.15**	9.15	6.71
**308.15**	9.19	6.73
**313.15**	9.23	6.76
**318.15**	9.27	6.78
**323.15**	9.32	6.80
**328.15**	9.36	6.83
**333.15**	9.40	6.85
**338.15**	9.45	6.87
**343.15**	9.49	6.90
**348.15**	9.54	6.92
**353.15**	9.58	6.94

Table S4. Measured viscosity, (η, mPa.s), of the $[C_2mim][CF_3BF_3]$ and $[C_2mim][CF_3SO_2C(CN)_2]$ ILs at atmospheric pressure.

T(K)	η (mPa.s)	
	$[C_2mim][CF_3BF_3]$	$[C_2mim][CF_3SO_2C(CN)_2]$
**293.15**	29.47	30.35
**298.15**	25.59	25.00
**303.15**	21.90	20.90
**308.15**	19.25	17.71
**313.15**	16.90	15.19
**318.15**	14.73	13.17
**323.15**	13.10	11.53
**328.15**	11.71	10.17
**333.15**	10.52	9.05
**338.15**	9.41	8.10
**343.15**	8.62	7.30
**348.15**	7.86	6.61
**353.15**	7.18	6.02

Table S5. Gas Permeability[a] (P). Diffusivity (D) and Solubility (S) of the $[C_2mim][CF_3BF_3]$ and $[C_2mim][CF_3SO_2C(CN)_2]$ ILs ILs at 308.15K and 1 bar of feed pressure.

		IL Sample	
**Permeation Property**	**Gases**	**$[C_2mim][CF_3BF_3]$**	**$[C_2mim][CF_3SO_2C(CN)_2]$**
Permeability (barrer)	**CO_2**	706 ± 9	1424 ± 6
	**CH_4**	41.7 ± 0.9	167 ± 4
	**N_2**	20.3 ± 0.6	57.9 ± 0.7
D x 10^{12} (m^2/s)	**CO_2**	407 ± 3	672 ± 14
	**CH_4**	492 ± 25	2745 ± 313
	**N_2**	692 ± 4	265 ± 6
S x 10^6 (m^3 (STP) m^{-3} Pa^{-1})	**CO_2**	13.0 ± 0.1	16.0 ± 0.4
	**CH_4**	0.64 ± 0.04	0.46 ± 0.05
	**N_2**	0.22 ± 0.01	1.60 ± 0.02

[a] Barrer (1 Barrer = 10^{-10} cm^3 (STP) cm cm^{-2} s^{-1} $cmHg^{-1}$)
